# Hybrid Li/Na Ion Batteries: Temperature-Induced Reactivity of Three-Layered Oxide (*P*3-Na_2/3_Ni_1/3_Mg_1/6_Mn_1/2_O_2_) Toward Lithium Ionic Liquid Electrolytes

**DOI:** 10.3389/fchem.2020.600140

**Published:** 2020-11-20

**Authors:** Mariya Kalapsazova, Krassimir Kostov, Ekaterina Zhecheva, Radostina Stoyanova

**Affiliations:** Institute of General and Inorganic Chemistry, Bulgarian Academy of Sciences, Sofia, Bulgaria

**Keywords:** layered oxides, oxide surface, ionic liquid electrolyte, XPS analysis, hybrid metal ion batteries, storage performance at elevated temperatures, sodium ion batteries

## Abstract

Hybrid metal ion batteries are perceived as competitive alternatives to lithium ion batteries because they provide better balance between energy/power density, battery cost, and environmental requirements. However, their cycling stability and high-temperature storage performance are still far from the desired. Herein, we first examine the temperature-induced reactivity of three-layered oxide, *P*3-Na_2/3_Ni_1/3_Mg_1/6_Mn_1/2_O_2_, toward lithium ionic liquid electrolyte upon cycling in hybrid Li/Na ion cells. Through *ex situ* X-ray diffraction (XRD) and X-ray photoelectron spectroscopy (XPS) analyses, the structural and surface changes in *P*3-Na_2/3_Ni_1/3_Mg_1/6_Mn_1/2_O_2_ are monitored and discussed. Understanding the relevant changes occurring during dual Li^+^ and Na^+^ intercalation into *P*3-Na_2/3_Ni_1/3_Mg_1/6_Mn_1/2_O_2_ is of crucial importance to enhance the overall performance of hybrid Li/Na ion batteries at elevated temperatures.

## Introduction

Nowadays, hybrid metal ion batteries (HMIBs) are perceived as competitive alternatives to lithium ion batteries (LiIBs) because they provide better balance between energy/power density, battery cost, and environmental requirements (Yao et al., 2016; Whittingham et al., 2018; Stoyanova et al., 2019). In comparison with LiIBs, two types of charge carriers (such as Li^+^/Na^+^, Li^+^/Mg^2+^, Na^+^/Mg^2+^, etc.) participate in the electrochemical reaction at HIMBs instead of the single Li^+^ ions (Yao et al., 2016; Stoyanova et al., 2019). There are two operating mechanisms, according to which the energy is stored either by two separate redox reactions occurring at the one and the other electrode, or by co-intercalation/co-deintercalation of two charge carriers at each electrode (Yagi et al., 2014; Yao et al., 2016; Zhang et al., 2018; Kravchyk et al., 2019; Stoyanova et al., 2019). When the battery operation includes two separate redox reactions, the amount of electrolyte needs to be large in order to supply enough charge carriers upon cell charging and discharging, resulting in a penalty in energy density (Cheng et al., 2016). Experimentally, it has been found that this type of HIMBs displays undesired performance in terms of low capacity and limited electrochemical window (Cheng et al., 2016). These drawbacks can be overcome by elaboration of electrode materials with specific structure and composition, which ensure the intercalation of both charge carriers (Cho et al., 2014; Stoyanova et al., 2019). The co-intercalation of ions with different charges, ionic radii, and ion coordination can be suggested to trigger a series of competitive reactions in solid-state matrices leading to avoiding the sluggish kinetics and/or polarization effects during the diffusion of large monovalent and/or polyvalent ions (Stoyanova et al., 2019; Wang et al., 2019). Supporting this, it has experimentally been demonstrated that the co-intercalation of solvated ions facilitates their solid-state diffusion by reducing the ion–host interactions (Li et al., 2018). Through interlayer expansion, both theoretical and experimental data indicate an improvement of the intercalation kinetics of large and/or polyvalent ions (Shuai et al., 2016). To take an advantage of HMIBs over LiIBs, there is a strong need to define new electrode materials that have structural matrices ensuring an effective mobility of two types of ions.

Among several oxide and polyanionic compounds, sodium-deficient nickel-manganese oxides, Na_x_Ni_1/2_Mn_1/2_O_2_ (0.5 ≤ *x* < 0.75), with a three-layer stacking are positioned as most suitable positive electrodes in hybrid Li/Na ion cells (Kalapsazova et al., 2014). Because of the specific structure and redox affinity of nickel ions, Na_x_Ni_1/2_Mn_1/2_O_2_ are capable to intercalate both smaller Li^+^ and bigger Na^+^ ions, a property unavailable for the oxide with a two-layer stacking (Kalapsazova et al., 2015). In hybrid Li/Na ion cell, the *P*3-phase delivers a higher capacity at lower rates, whereas in sodium half-cell *P*3-Na_*x*_Ni_0.5_Mn_0.5_O_2_ exhibits a better rate capability (Kalapsazova et al., 2015). The co-intercalation of Li^+^ and Na^+^ into *P*3-Na_x_Ni_1/2_Mn_1/2_O_2_ is accompanied by a structural transformation from *P*3 to *O*3, which, on its turn, determines furthermore the specific voltage profile of layered oxide and enables to reach a higher capacity in comparison with individual Na^+^ intercalation (Kalapsazova et al., 2015). However, the cycling stability of *P*3-Na_x_Ni_1/2_Mn_1/2_O_2_ is still far from the desired.

Another common issue concerning layered lithium- and sodium-containing oxides is their poor cycling stability at elevated temperatures (Wright et al., 2018; Zhou et al., 2020). From structural point of view, the high operating temperatures accelerate the structural evolution of lithium–manganese-rich layered oxides into core–shell structure during cycling along with lattice oxygen extraction and lattice densification, transition-metal migration, and aggregation on the crystal surface (Yu et al., 2018). The transition metal migration during cycling is also responsible for the voltage decay observed at lithium-rich oxides (Pham et al., 2016). The cationic redistribution process between layers has also been established for Mg-substituted *P*3-Na_x_Ni_1/2_Mn_1/2_O_2_ when used as an electrode in Na-ion cells (Kalapsazova et al., 2020). This process occurring during Na^+^ extraction is intensified at elevated temperatures and, contrary to the lithium-containing oxides, has a positive impact on the cycling stability (Kalapsazova et al., 2020).

The operating temperature affects not only the structure of electrode materials, but also the electrode/electrolyte interface (Hekmatfar et al., 2019). A diversity of reactions such as electrolyte decomposition, adsorption of decomposed products, and film growing on electrode surface proceed at the electrode–electrolyte interface (Yua and Manthiram, 2018), all of them being sensitive on the temperature of cell cycling. Thus, the storage performance of electrodes at elevated temperature depends critically on the type of electrolyte used; because of the higher thermal stability, the ionic liquid (IL) electrolytes are preferred over the conventional carbonate-based electrolytes (Oltean et al., 2018). Although the temperature-induced structural and surface changes of the electrode materials upon individual Li^+^ and Na^+^ intercalation are examined intensively in regard of the practical application of Li and Na ion batteries (Wright et al., 2018; Zhou et al., 2020), the dual-ion intercalation issue under elevated temperatures remains unclear in hybrid Li/Na ion batteries.

Herein, we first examine the temperature-induced reactivity of three-layered oxide, *P*3-Na_2/3_Ni_1/3_Mg_1/6_Mn_1/2_O_2_, toward lithium IL electrolyte upon cycling in hybrid Li/Na ion cells. The Mg-substituted oxide is selected as it is designed to work in sodium ion cells at elevated temperatures (Kalapsazova et al., 2020). Through *ex situ* XRD and XPS analyses, the structural and surface changes in *P*3-Na_2/3_Ni_1/3_Mg_1/6_Mn_1/2_O_2_ are monitored. Understanding the relevant changes occurring during dual Li^+^ and Na^+^ intercalation into *P*3-Na_2/3_Ni_1/3_Mg_1/6_Mn_1/2_O_2_ is of crucial importance to enhance the overall performance of hybrid Li/Na ion batteries at elevated temperatures.

## Experimental Section

### Synthetic Procedures

Sodium–nickel–magnesium–manganese oxide, *P*3-Na_2/3_Ni_1/3_Mg_1/6_Mn_1/2_O_2_, was prepared by using freeze-dried acetate precursors. The preparation procedure was reported elsewhere (Kalapsazova et al., 2020). The homogeneous acetate precursors were obtained by freeze drying of the corresponding Na, Ni, Mg, and Mn acetate solutions (0.5 M). The acetate precursors are decomposed at 400°C in O_2_ atmosphere for 3 h, followed by homogenization, palletization, and annealing at 700°C in air for 24 h. The structure and the elemental composition of the oxide were checked via XRD and ICP-OES.

### Electrode Preparation and Cell Assembly

The electrode slurry consists of 85 wt% active material, 10 wt% carbon black super C65 (TIMCAL/IMERYS), and 5 wt% polyvinylidene fluoride (Solef 6020, Solvay Polymer Specialties) dissolved in N-methyl-2-pyrrolidone (10% solution). The mixture of all components was ball milled, and the slurry was cast on aluminum foil, followed by drying at 60°C overnight. The disk electrodes with a diameter of 12 mm were cut, pressed, and dried at 120°C under vacuum. The mass loading of active material varied between 3.38 and 3.91 mg cm^−2^.

Three electrode Swagelok cells were assembled in an argon-filled glove box with water and oxygen content of <0.1 ppm. The counter and reference electrode consisted of a clean lithium metal disk. Two types of electrolyte solutions were applied: (i) conventional lithium electrolyte consisting of 1M LiPF_6_ in EC:DEC (1:1 vol/vol) (Sigma–Aldrich, battery grade), and (ii) the IL-based electrolyte comprising LiTFSI (99.5%, 3 M) in Pyr_14_FSI (synthesized in-house) in 1:9 molar ratio. The water content in IL-based electrolyte was lower than 6 ppm as from checked Karl Fischer titration (Mettler–Toledo); 140 μL of electrolytes was soaked in glass microfiber separators (Whatman GF/D).

### Characterization of the Materials

The electrochemical characterization was carried out in galvanostatic mode between 2.0–4.3 V and 2.0–4.5 V. All tests were performed in climatic chambers at a temperature of 20, 40, and 60 ± 2°C. To ensure the accuracy of the measurement, each test is repeated at least twice. After cycling, the electrochemical cells were disassembled inside a glove box, followed by removing and washing of the working electrodes with dimethyl carbonate (DMC) to eliminate electrolyte residues. The *ex situ* powder X-ray diffraction was performed on Bruker Advance D8 powder diffractometer with a LynxEye detector by using CuKα-radiation. The electrode surface was analyzed by X-ray photoelectron spectrometer (AXIS Supra, Kratos Analitycal Ltd.) with monochromatic AlKα source (1,486.6 eV) and charge neutralization system. The photo-emitted electrons were separated, according to their kinetic energy, by a 180°-hemispherical analyzer having a total instrumental resolution of 0.54 eV (as it was measured by the full-width half-maximum of Ag 3d_5/2_ line) at pass energy of 20 eV. The binding energies were determined with an accuracy of ±0.05 eV. The curve profiles of the XPS spectra were analyzed using a symmetrical Gaussian-Lorentzian curve fitting after Shirley's-type subtraction of the background. The commercial data-processing software of Kratos Analytical Ltd. was employed for the calculation of elemental concentrations in atomic percent.

## Results and Discussions

### Lithium Storage of *P*3-Na_2/3_Ni_1/3_Mg_1/6_Mn_1/2_O_2_ at Elevated Temperatures

**Figure 1:**
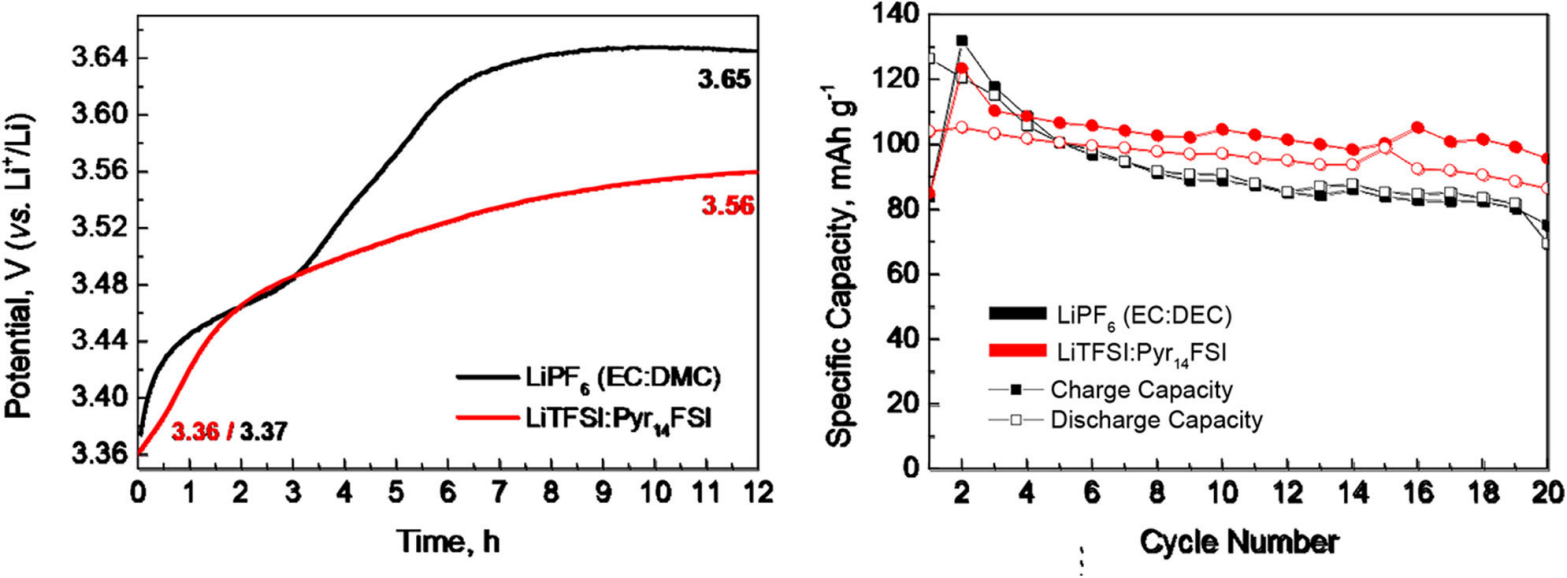
**(Left)** Time evolution of OCV for *P*3-Na_2/3_Ni_1/3_Mg_1/6_Mn_1/2_O_2_ in hybrid ion cell employing carbonate- and IL-based electrolyte at 20°C. **(Right)** Lithium storage capacity of *P*3-Na_2/3_Ni_1/3_Mg_1/6_Mn_1/2_O_2_ determined at 20°C in carbonate- and IL-based electrolytes.

The lithium storage capacity in *P*3-Na_2/3_Ni_1/3_Mg_1/6_Mn_1/2_O_2_ is determined in hybrid Li/Na cell using carbonate- and IL-containing electrolyte (Figure 1). Prior to the cell cycling, the electrode is soaked in both electrolytes for 12 h (Figure 1). As one can see, the open-circuit voltage (OCV) increases after several hours of soaking and reaches a stable value after 12th hour. The comparison shows that OCV increases more in the carbonate-based electrolyte (i.e., in 1 M LiPF_6_ in EC/DMC). This indicates that some interactions between electrode and electrolyte (such as partial exchange of Na^+^ with Li^+^ and/or Na^+^ extraction) take place. Under these conditions, it appears that the interaction of the oxide with carbonate-based electrolyte is more significant. Furthermore, *P*3-Na_2/3_Ni_1/3_Mg_1/6_Mn_1/2_O_2_ delivers slightly higher specific capacity in IL electrolyte than that in the carbonate-based electrolyte (Figure 1). Taking into account these results, as well as the high thermal stability of IL, the electrochemical behavior of *P*3-Na_2/3_Ni_1/3_Mg_1/6_Mn_1/2_O_2_ is examined at elevated temperatures only in IL electrolyte.

**Figure 2:**
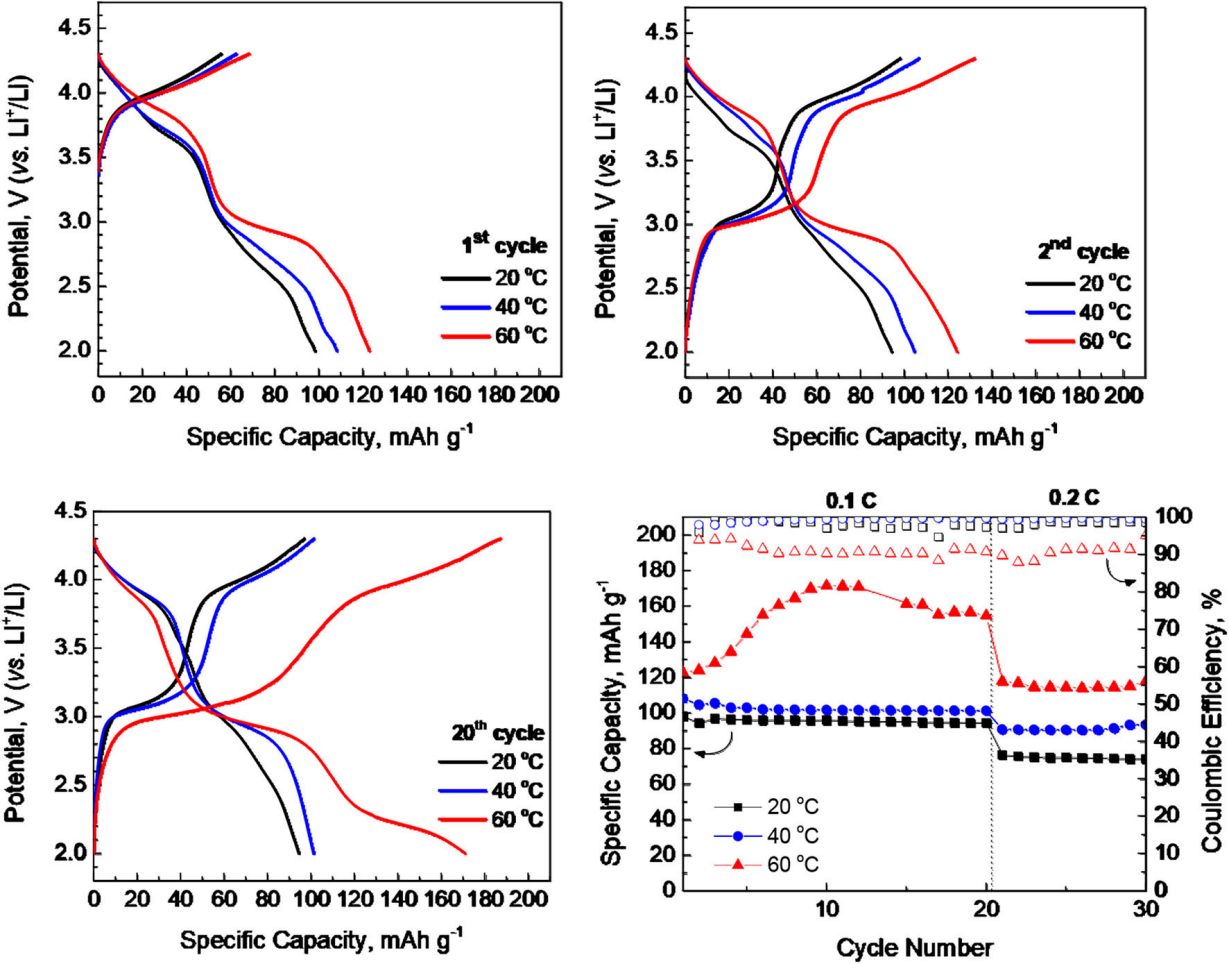
Charge and discharge curves after the 1st, 2nd, and 20th cycle for *P*3-Na_2/3_Ni_1/3_Mg_1/6_Mn_1/2_O_2_ working in IL electrolytes at 20, 40, and 60°C. Rate capability of *P*3-Na_2/3_Ni_1/3_Mg_1/6_Mn_1/2_O_2_ at 20, 40, and 60°C (bottom, right).

Figure 2 compares the lithium storage properties of *P*3-Na_2/3_Ni_1/3_Mg_1/6_Mn_1/2_O_2_ at 20, 40, and 60°C. By increasing the operating temperature, there is a continuous increase in the lithium capacity. The temperature-induced increase in the lithium storage capacity becomes higher after prolonged cycling (Figure 2). Although the capacity delivered from *P*3-Na_2/3_Ni_1/3_Mg_1/6_Mn_1/2_O_2_ at 20 and 40°C is sustained between the 1st and 20th cycle, the capacity at 60°C increases smoothly up to the 10th cycle, and after which, a relatively constant value is achieved (Figure 2). The changes in the magnitude of capacity are accompanied with corresponding alteration in the charge/discharge curve profiles (Figure 2). At 20°C, the charge and discharge curves consist of two voltage plateaus at 3.0 and 3.9 V. The same voltage plateaus have been observed when *P*3-Na_2/3_Ni_1/3_Mg_1/6_Mn_1/2_O_2_ cycled in carbonate-based electrolyte (Kalapsazova et al., 2015). By increasing the operating temperature from 20 to 60°C, the low-voltage plateau (i.e., at 3.0 V) is extended, whereas the high-voltage plateau is preserved intact. In addition, a new discharge plateau at 2.2 V grows upon prolonged cycling at 60°C. This implies that significant structural changes occur when *P*3-Na_2/3_Ni_1/3_Mg_1/6_Mn_1/2_O_2_ cycled at 60°C.

The operating temperature has also an effect on the rate capability of *P*3-Na_2/3_Ni_1/3_Mg_1/6_Mn_1/2_O_2_. The best rate capability is observed at 40°C, while at 60°C, there is a higher capacity loss going from 0.1 to 0.2 C. Even in this case, the capacity at 60°C is still higher than that determined at 20 and 40°C. However, the Coulombic efficiency at 60°C is worse in comparison with that determined at 20 and 40°C. This can be related with the corrosion of aluminum collector caused by LiTFSI salt (Kühnel et al., 2012; Matsumoto et al., 2013). The corrosion comprises the formation of Al-TFSI complexes on the Al surface, which exhibit different solubility in carbonate- and IL-based solvent and as a consequence—different corrosion affinities. Because of the temperature dependence of the solubility, it can be suggested that the corrosion is intensified at 60°C. For that reason, the electrochemical behavior of *P*3-Na_2/3_Ni_1/3_Mg_1/6_Mn_1/2_O_2_ at 20 and 40°C is examined in broader potential range by extending the upper potential limit from 4.3 to 4.5 V and keeping the lower potential limit at 2.0 V.

**Figure 3:**
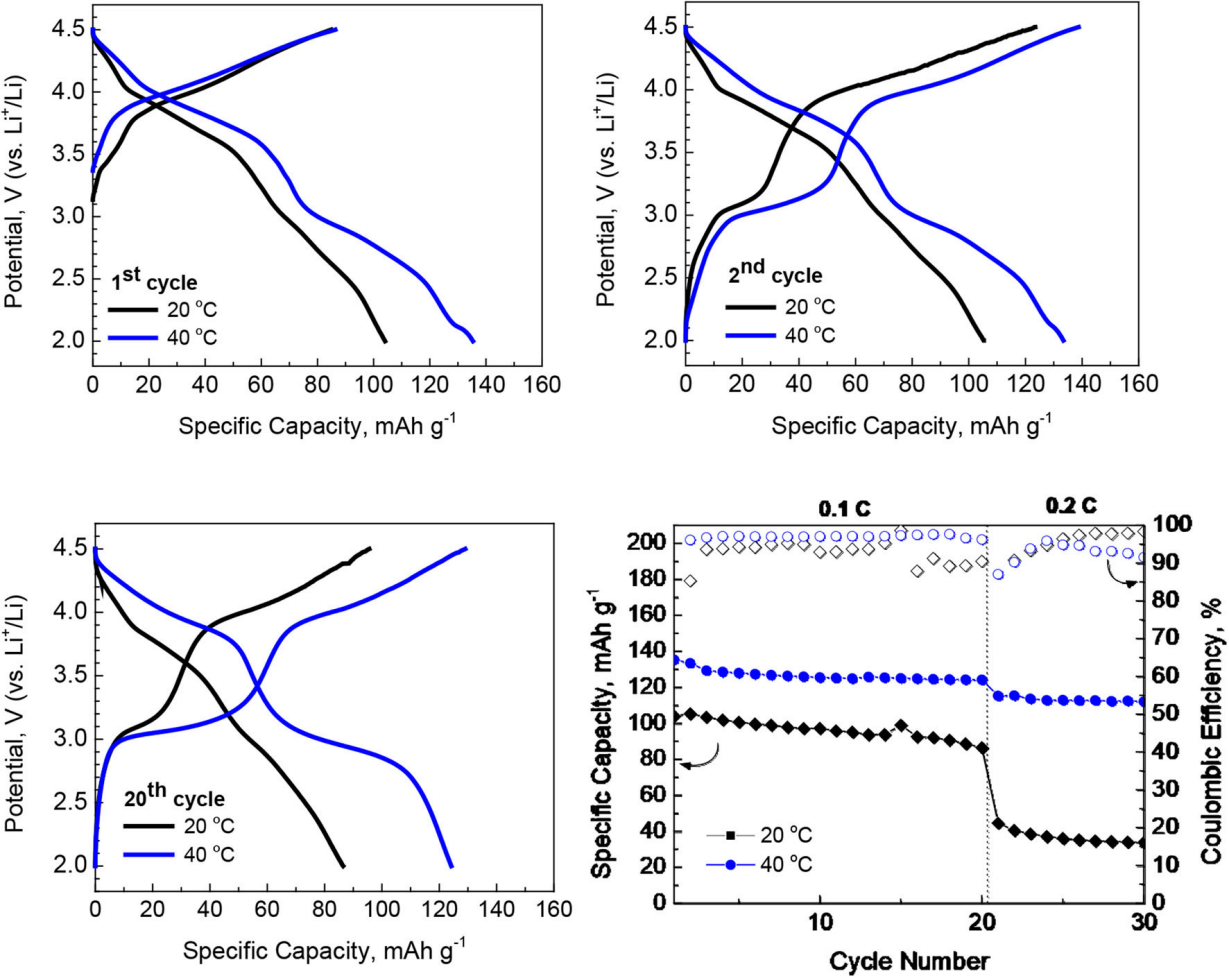
Charge and discharge curves after 1st, 2nd, and 20th cycle for *P*3-Na_2/3_Ni_1/3_Mg_1/6_Mn_1/2_O_2_ working at 20 and 40°C in extended potential range of 2.0–4.5 V. Rate capability of *P*3-Na_2/3_Ni_1/3_Mg_1/6_Mn_1/2_O_2_ at 20 and 40°C (bottom, right).

The electrochemical performance of *P*3-Na_2/3_Ni_1/3_Mg_1/6_Mn_1/2_O_2_ in extended potential range is given on Figure 3. The main trend for increasing the capacity by rising the operating temperature from 20 to 40°C is observed again. It is noticeable that the higher capacity is achieved without alteration in the charge/discharge curve profiles: there are two well-resolved plateaus at 3.0 and 3.9 V especially after prolonged cycling as in the case of narrower potential range between 2.0 and 4.3 V (Figure 2). The most important finding is the excellent rate capability of *P*3-Na_2/3_Ni_1/3_Mg_1/6_Mn_1/2_O_2_ at 40°C even in extended potential range, where the higher capacity is reached: at 0.2°C, the capacity of around 130 mAh/g between 2.0 and 4.5 V vs. 95 mAh/g between 2.0 and 4.3 V.

**Figure 4:**
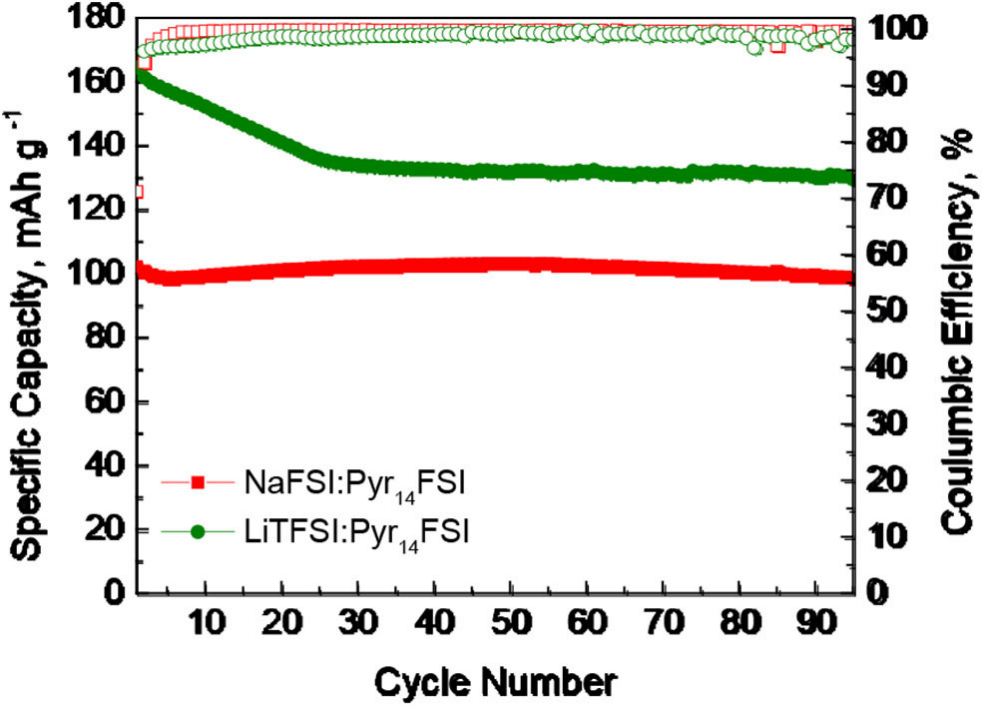
Cycling stability test at 40°C for *P*3-Na_2/3_Ni_1/3_Mg_1/6_Mn_1/2_O_2_ cycled in sodium and hybrid Li/Na ion cells using sodium and lithium IL electrolyte.

Recently, we have demonstrated that, in sodium ion cell using sodium IL electrolyte (such as NaFSI in Pyr_14_FSI in the molar ratio 1:9), *P*3-Na_2/3_Ni_1/3_Mg_1/6_Mn_1/2_O_2_ displays also better rate capability at 40°C (Kalapsazova et al., 2020). In this study, the cycling stabilities at 40°C for three-layered oxide in sodium and hybrid Li/Na cells are compared (Figure 4). In sodium ion cell, *P*3-Na_2/3_Ni_1/3_Mg_1/6_Mn_1/2_O_2_ delivers a capacity of around 100 mAh/g with an excellent cycling stability due to the reversible Na^+^ intercalation. In hybrid Li/Na ion cell, the capacity decreases up to the 20th cycle reaching a magnitude of around 135–140 mAh/g that is stable after that. The higher capacity of *P*3-Na_2/3_Ni_1/3_Mg_1/6_Mn_1/2_O_2_ in hybrid ion cell is a consequence of the co-intercalation of Na^+^ and Li^+^ ions. This result discloses that *P*3-Na_2/3_Ni_1/3_Mg_1/6_Mn_1/2_O_2_ is best suited to be a positive electrode material for hybrid ion batteries.

### Temperature-Induced Changes in the Structure of *P*3-Na_2/3_Ni_1/3_Mg_1/6_Mn_1/2_O_2_

**Figure 5:**
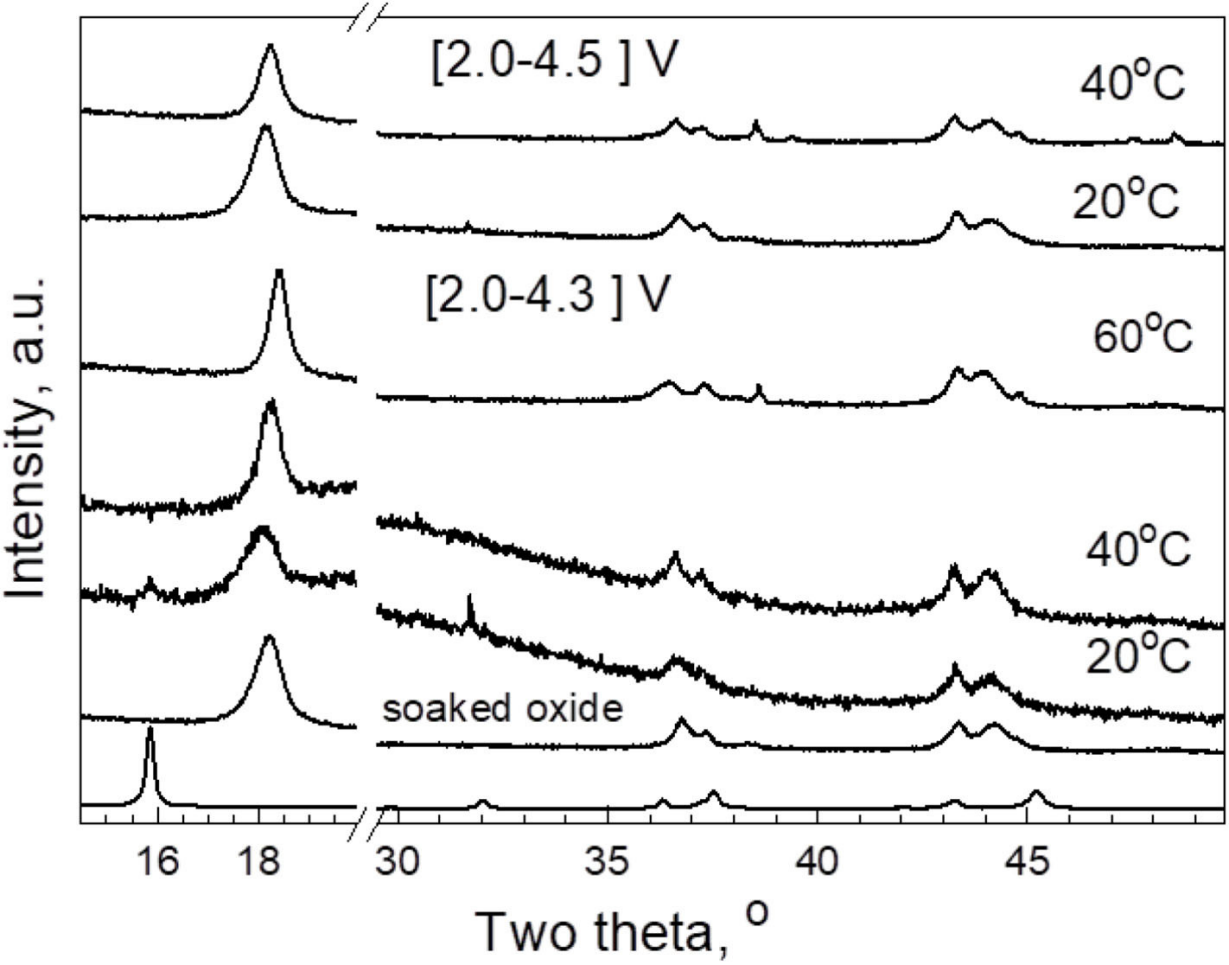
XRD patterns of pristine *P*3-Na_2/3_Ni_1/3_Mg_1/6_Mn_1/2_O_2_, oxide soaked in IL electrolyte for 12 h, and discharged electrodes cycled at 20, 40, and 60°C in LiTFSI-Pyr_14_FSI between 4.3 and 2.0 V and between 4.5 and 2.0 V.

To rationalize the structural stability of *P*3-Na_2/3_Ni_1/3_Mg_1/6_Mn_1/2_O_2_ after co-interaction of Li^+^ and Na^+^, *ex situ* XRD measurements were carried out (Figure 5). The subjects of study are electrodes cycled at 20, 40, and 60°C in LiTFSI-Pyr_14_FSI between 4.3 and 2.0 V for 30 cycles and recovered from cells in the discharged state (2.0 V). For comparison, the electrodes cycled at 20 and 40°C in extended potential range between 4.5 and 2.0 V were also analyzed. In addition, the same figure shows the XRD pattern of *P*3-Na_2/3_Ni_1/3_Mg_1/6_Mn_1/2_O_2_ soaked in the IL electrolyte for 12 h prior to the cell cycling. In comparison with pristine oxide Na_2/3_Ni_1/3_Mg_1/6_Mn_1/2_O_2_ having *P*3 type of structure, the XRD patterns of soaked oxide and electrodes are indexed in a structural model based on the *O*3 type of structure. The difference between *P*3 and *O*3 type of structure reflects the stacking arrangement of layers in the unit cell, which, on its turn, is responsible for the transformation of the coordination of alkali ions from prismatic to octahedral ones. Although Na^+^ ions occupy both the prismatic and octahedral sites, the Li^+^ ions reside only the octahedral sites. Thus, the transformation of the *P*3- into *O*3-type structure is a mark for occurrence of Li^+^ ions in desodiated oxides.

The lattice parameters are listed on Table 1. After oxide soaking, both *a* and *c* parameters are contracted. This means that Na^+^ ions are extracted from *P*3-Na_2/3_Ni_1/3_Mg_1/6_Mn_1/2_O_2_, the charge compensation being attained by oxidation of transition metal ions. The highly oxidized ions possess smaller ionic radii, as a result of which the *a* parameter (that expresses the intralayer distance between ions) decreases. In THE same order, the *c* parameter also decreases, which is related to an insertion of smaller Li^+^ ions in the interlayer space of *P*3-Na_2/3_Ni_1/3_Mg_1/6_Mn_1/2_O_2_ instead of bigger Na^+^ ions. This is another proof that layered oxide interacts with IL electrolyte prior to the cell cycling. It is of importance that the lattice parameters of soaked oxide deviate from that of lithium nickel-manganese oxide, LiNi_1/2_Mn_1/2_O_2_, obtained by solid-state reaction (Yoncheva et al., 2009): *a* = 2.8578 and *c* = 14.479 vs. *a* = 2.8788 Å and *c* = 14.2819 Å, respectively. This means that mixed Li, Na oxides are obtained after the electrochemical reaction in lithium IL electrolyte. The formation of mixed Li, Na oxides with an *O*3 type of structure can be understood on the basis of available crystallographic data for layered oxides. For example, the structure of layered cobaltates Li_~0.42_Na_~0.37_CoO_2_ is composed of two alternative AO_2_ blocks: a *P2*-type sodium block and an *O3*-type lithium one (Bos et al., 2007; Berthelot et al., 2011). Contrary to Li_~0.42_Na_~0.37_CoO_2_, the incorporation of Li into *O3*-NaNi_0.5_Mn_0.5_O_2_ takes place through the formation of a *P2/O3* intergrowth at an atomic scale (Lee et al., 2014). In analogy, the structure of mixed Na^+^/Li^+^-nickel-manganese oxides (i.e., *O*3-Li_y_Na_(0.1−0.2)_Ni_0.5_Mn_0.5_O_2_) obtained from *P*3-Na_2/3_Ni_1/2_Mn_1/2_O_2_ is also described as composed of *P3* and *O3* type of domains (Kalapsazova et al., 2019).

**Table 1:** Lattice parameters of pristine *P*3-Na_2/3_Ni_1/3_Mg_1/6_Mn_1/2_O_2_, soaked oxide and discharged electrodes cycled at 20, 40, and 60°C in narrow (2.0–4.3 V) and broad (2.0–4.5 V) potential range.

**Conditions**	***a*, Å**	***c*, Å**
Pristine oxide	2.8889 ± 0.0001	16.7789 ± 0.0019
Oxide soaked in IL electrolyte for 12 h	2.8578 ± 0.0006	14.479 ± 0.009
Discharged electrode at 2.0 V, 20°C, range of 4.3–2.0 V	2.8753 ± 0.0004	14.832 ± 0.006
Discharged electrode at 2.0 V, 40°C, range of 4.3–2.0 V	2.8791 ± 0.0012	14.635 ± 0.012
Discharged electrode at 2.0 V, 60°C, range of 4.3–2.0 V	2.8839 ± 0.0008	14.399 ± 0.009
Discharged electrode at 2.0 V, 20°C, range of 4.5–2.0 V	2.8601 ± 0.0011	14.639 ± 0.029
Discharged electrode at 2.0 V, 40°C, range of 4.5–2.0 V	2.8691 ± 0.0006	14.569 ± 0.019

**Figure 6:**
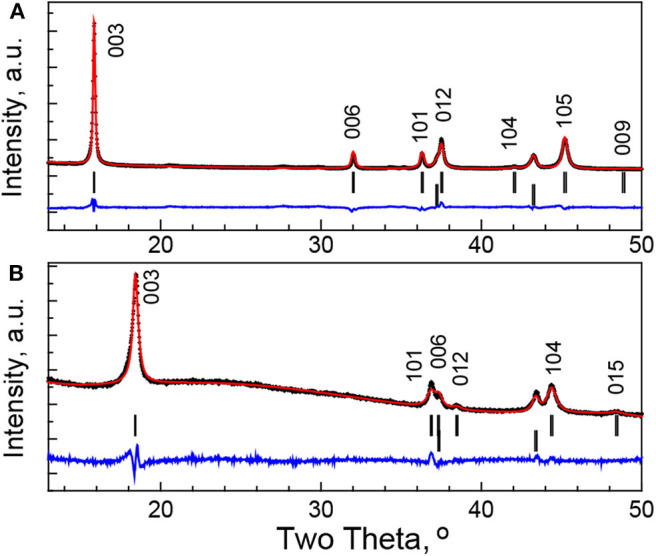
XRD patterns and simulated ones using Rietveld refinement for pristine *P*3-Na_2/3_Ni_1/3_Mg_1/6_Mn_1/2_O_2_ **(A)** and soaked oxide in IL electrolyte for 12 h **(B)**. The Bragg's reflections for *P*3 and O3 types of structure and the impurity of NiO-like phase are given.

In order to give rough evaluation of the amount of Na^+^ in soaked oxide, a Rietveld analysis is also performed (Figure 6). However, these data should be taken with caution because of a broadening of the diffraction peaks (Figure 5). Keeping the Mg content and using the same isotropic thermal factors as in the case of the pristine oxide, the refinement gives the following site occupancies for Li and Na: 0.31 and 0.14, respectively. This reveals the decrease in the amount of Na^+^ in the soaked oxide in respect of Li^+^ ions.

After the electrochemical reaction, the lattice parameters undergo further changes (Table 1). By increasing the operating temperature, there is a strong decrease in the *c* parameter, but the *a* parameter is slightly increased. In addition, the *a* parameter of discharged oxide tends toward the *a* parameter of the pristine oxide. After the extension of the potential range, the *c* parameter decreases furthermore. The temperature-induced contraction in the *c* parameter can be attributed to a cationic exchange process leading to an appearance of Ni^2+^ and/or Mg^2+^ ions with close ionic radii in the interlayer space and, *vice versa*, Li^+^ in the transition metal layers: the higher the amount of Ni^2+^ and/or Mg^2+^ ions, the lower is the degree of the trigonal lattice distortion, and smaller is the magnitude of the *c* parameter. This is a well-established phenomenon for nickel-rich lithium oxides, where Ni^2+^ from transition-metal layers are exchanged with Li^+^ from interlayer space due to their close ionic radii (Zhecheva and Stoyanova, 1993). Recently, we have demonstrated that Na^+^ extraction from *P*3-Na_2/3_Ni_1/3_Mg_1/6_Mn_1/2_O_2_ at potentials higher than 4.2 V vs. Na^+^/Na unlocks a reversible reaction of transfer of Mg^2+^ and Ni^2+^ ions from the transition metal layer to the Na^+^-depleted interlayer slab (Kalapsazova et al., 2020). It is worth to mention that Na^+^ ions (contrary to Li^+^ ones) cannot be inserted into the transition metal layers due to their bigger sizes. Here, the *ex situ* XRD analysis reveals that the cationic exchange is initiated after Li^+^,Na^+^ co-intercalation into *P*3-Na_2/3_Ni_1/3_Mg_1/6_Mn_1/2_O_2_, and it is intensified with raising of the operating temperature and with extension of the potential range. Under these conditions of cycling, *P*3-Na_2/3_Ni_1/3_Mg_1/6_Mn_1/2_O_2_ is transformed into mixed Li,Na oxide with *O*3-type structure and cationic distribution that mimics the cationic distribution in lithium-rich oxides (i.e., Li_1+x_TM_1−x_O2). The temperature-induced structural and composition transformation is related with an enhanced storage performance of *P*3-Na_2/3_Ni_1/3_Mg_1/6_Mn_1/2_O_2_ (Figures 2, 3).

### Surface Reactivity of *P*3-Na_2/3_Ni_1/3_Mg_1/6_Mn_1/2_O_2_ Toward Lithium IL Electrolyte

**Figure 7:**
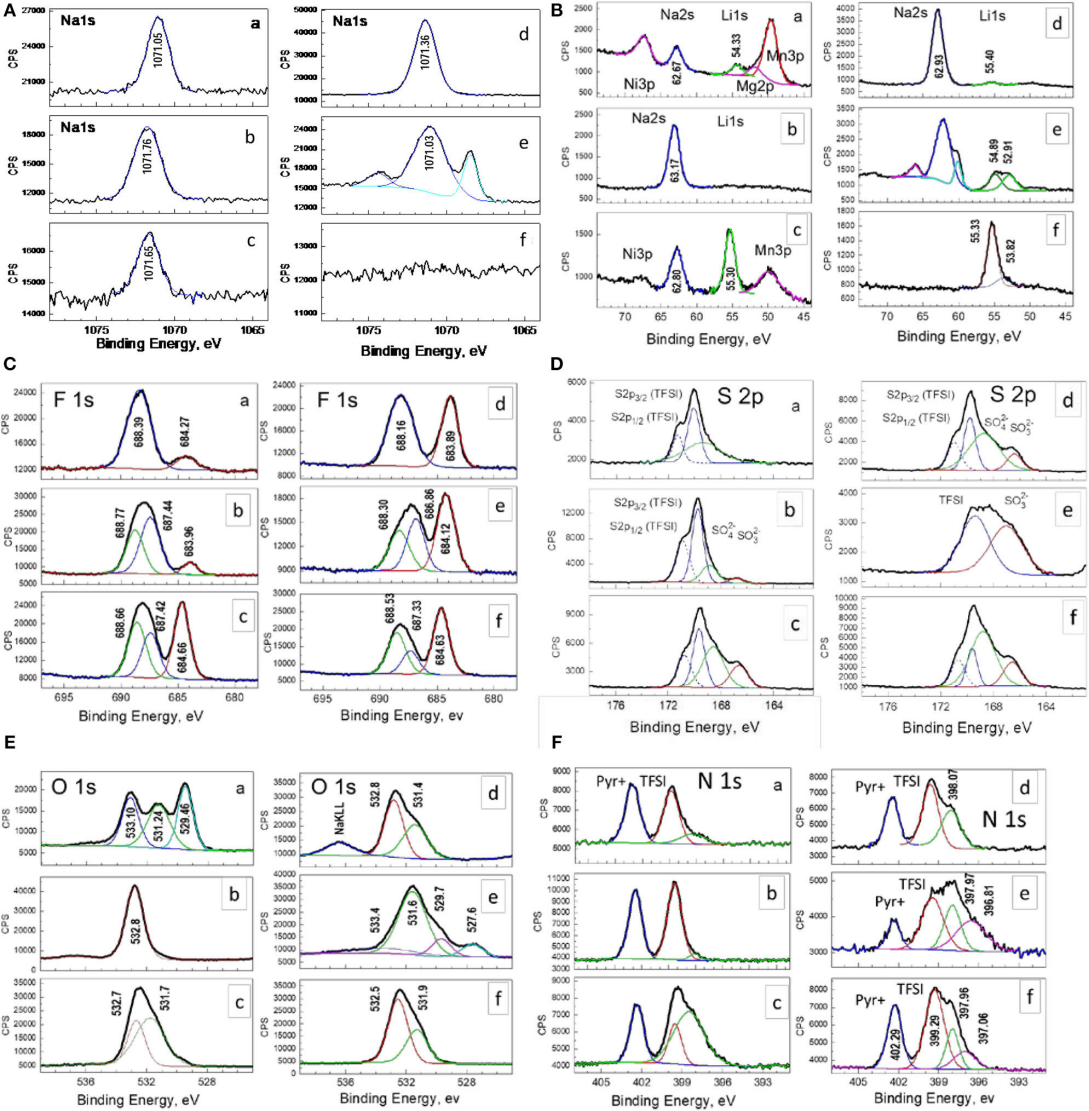
XPS spectra in the energy region of Na 1s, Na 2s, Li1s, F 1s, S 2p, O 1s, and N 1s for soaked oxide **(A)** and electrodes cycled at 20 **(B)** and 40°C **(C)** in the potential range of 2.0–4.5 V. The electrode cycled at 20 **(D)**, 40 **(E)**, and 60°C **(F)** in the range of 2.0–4.3 V are also shown.

The electrode surface is probed by XPS spectroscopy. Figure 7 gives the XPS spectra of soaked oxide and discharged electrodes in the energy region of Na 1s, Na 2s, Li1s, F 1s, S 2p, O 1s, and N 1s. The peak identification is based on previous data for NaF, PVdF, FSI^−^, and Pyr_14_ (Erdem et al., 2001; Eshetu et al., 2016, 2019; Do et al., 2019; Liu et al., 2019).

In the Na1s and Na2s regions, the XPS spectra display symmetric peaks, whose centers of gravity being relatively constant for soaked oxide and electrodes cycled at different operating temperatures and potential ranges (Figure 7). These Na1s and Na2s peaks can be assigned to Na atoms in NaF (Brisson et al., 2006). In agreement with this assignment, the F 1 s spectrum shows a peak at around 684 eV typical for F atoms in NaF (Kalapsazova et al., 2014, 2017). In comparison with the case of Na 1s and Na 2s, the F 1s peak shows, however, a tendency to increase from 683.71 to 684.27 eV with enhancing the operating temperature irrespective of the potential range of cycling. In addition, by increasing the operating temperature, a new signal in the range of Li 1s binding energy grows in intensity. The new peak centered at around 55 eV corresponds to Li atoms in LiF. This indicates that, at elevated temperatures, LiF is preferentially deposed on the electrode surface in comparison with NaF. Supporting this suggestion, Table 2 lists the relative ratio between Na2s and Li 1s. The use of Na2s and Li1s allows comparing the amounts of Li and Na at comparable depths from the electrode surface.

**Table 2:** Surface composition for soaked and cycled electrodes determined by XPS analysis.

**Sample**	**Na2s/Li1s**	**Li/S**	**Na/S**	**F/S**	**O/S**	**N/S**
Soaked oxide	0.6	0.8	0.4	3.2	5.9	1.7
Oxide cycled at 20°C between 2.0 and 4.3 V	5.0	0.2	1.1	1.8	2.0	0.9
Oxide cycled at 40°C between 2.0 and 4.3 V	0.6	4.9	2.8	2.7	7.4	1.0
Oxide cycled at 60°C between 2.0 and 4.3 V	0.0	1.8	0	1.8	2.3	1.0
Oxide cycled at 20°C between 2.0 and 4.5 V	5.3	0.1	0.4	1.7	1.8	0.9
Oxide cycled at 40°C between 2.0 and 4.5 V	0.1	1.3	0.1	1.7	2.3	0.9

The analysis of Na1s, Na2s, F1s, and Lis spectra of soaked oxide unveils that before the electrochemical reaction the electrode surface is also covered with LiF/NaF. This result, along with XRD data, indicates that interaction between the oxide and electrolyte during oxide soaking encompasses both the bulk and surface of electrode. The close inspection of the XPS survey in the binding-energy region from 80 to 40 eV reveals that, only for the soaked electrode, there are three additional peaks attributed to Ni 3p, Mg 2p, and Mn 3p photoelectron emission (Figure 7), originating from the electrode material. Therefore, the thickness of the surface film comprising NaF/LiF should be less than the XPS analysis depth. In turn, this depth is determined approximately three times the inelastic mean free path of the photoemitted electrons in the solid. For the kinetic energies of Ni 3p-, Mg 2p-, and Mn 3p-photoelectrons (in the range of 1,400–1,440 eV), the inelastic mean free path is ~2 nm (Seah and Dench, 1979), which determines an analysis depth of about 6 nm. Therefore, the thickness of the NaF surface layer for the soaked electrode should be below 6 nm. On the other hand, the cycled electrodes do not display any signals due to Ni 3p, Mg 2p, and Mn 3p. This discloses that the surface film becomes thicker during the electrode cycling (i.e., more than 6 nm), which is a consequence of competitive processes of dissolution/deposition of alkaline fluorides. It is of importance that the competitive processes are sensitive toward the operating temperature. The fingerprints of these processes are the different binding environments detected in the Na1s, Na 2s, and Li 1s binding energies for electrode cycled at 40°C in a narrow potential range (Figure 7).

To check the stability of the electrolyte salt during cycling, the S2p energy region is examined (Figure 7). The spectra of soaked and cycled electrodes consist of two components, which can be related with spin-orbit splitting of S 2p3/2 and S 2p1/2 levels. These components originate from S atoms included in TFSI^−^ counterions. The detection of TFSI^−^ underlines that, before the electrochemical reaction, there is an adsorption of electrolyte salt without decomposition on the oxide surface. In addition, two peaks at around 166.5 and 168.5 eV are superimposed on the peak due to TFSI^−^. These peaks are too broad and the spin-orbit doublets are not resolved. Irrespective of this, the two peaks can be assigned to S atoms in sulfites/sulfonyls and sulfates, respectively. The appearance of sulfur-containing decomposed products is witnessed on the participation of the IL electrolyte in the surface film growth after oxide soaking, as well as upon electrode cycling (Figure 7). The deposition of sulfates and sulfites/sulfonyls is also detected by XPS spectra in the energy region of O 1s. The peaks typical for O atoms in Li_2_SO_4_ and Na_2_SO_4_ appear at around 532.6 and 532.0 eV (Fantauzzi et al., 2014). (It is worth to recall that Li and Na atoms in LiF/NaF and Li_2_SO_4_/Na_2_SO_4_ exhibit close binding energies). The O 1s spectrum of the soaked electrode displays additional peaks with binding energies corresponding to the lattice oxygen (i.e., 529.2 eV) and FSI^−^ counterion (i.e., 533 eV). This indicates that the surface film on soaked oxide is either thinner than that for cycled electrode. Upon electrode cycling, the relative part of the decomposed products becomes more and more with increasing the operating temperature. This trend is observed irrespective of the used potential range.

The presence of decomposed electrolyte products is further supported by the XPS spectra in the N 1s region. The spectra of all samples can be deconvoluted at least of three components; two of them correspond to electrolyte ions ${\text{Pyr}}_{14}^{+}$ and TFSI^−^, whereas the last peak is associated with nitride product (Figure 7). As in the case of sulfur-containing products, the relative part of the nitrogen-containing decomposed products increases with the operating temperature.

The changes in surface composition of cycled electrode are summarized on Table 2. The drastic variation in the film composition is associated with the amount of Li and Na species, whereas the rest O, F, S, and N species are relatively constant with exception of the soaked oxide. By increasing the operation temperature, the surface film with a thickness of around 6 nm becomes richer on Li^+^ species in expense of the Na^+^ ones. The slight variation in O, F, S, and N species reflects rather the adsorption of TFSI^−^ and Pyr^+^ components of the electrolyte. However, the fine analysis demonstrates that the sulfur- and nitrogen-containing decomposed products are much higher at elevated temperature than that at 20°C (Figure 6). All these correlations are valid irrespective of the used potential range (i.e., 2.0–4.3 and 2.0–4.5 V).

## Conclusions

In hybrid Li/Na ion cell, the function of *P*3-Na_2/3_Ni_1/3_Mg_1/6_Mn_1/2_O_2_ as an electrode material deviates from the ordinary operation in individual Na-ion batteries. This is a consequence of the reactivity of *P*3-Na_2/3_Ni_1/3_Mg_1/6_Mn_1/2_O_2_ toward the lithium IL electrolyte. This interaction begins before the electrochemical reaction, resulting in a transformation of *P*3 into *O*3 type of structure and an enrichment of the oxide surface with LiF. The *in situ*–generated *O*3 phase participates in the electrochemical reaction. Upon numerous Li^+^/Na^+^ intercalation, several competitive reactions encompassing both the bulk and surface of oxide are stimulated by the operating temperature and the used voltage window. By raising the operation temperature and by extending the voltage window, the exchange reaction of Ni^2+^,Mg^+^ from transition metal layers with Li^+^ from the interlayer slab is accelerated, yielding mixed Li, Na oxide with cationic distribution that mimics those in lithium-rich oxides (i.e., Li_1+x_TM_1−x_O_2_).

Along with the structural transformation, a film enriched with LiF, NaF, electrolyte-decomposed products (such as sulfur- and nitrogen-containing ones) grows on the oxide surface, reaching a thickness of more than 6 nm. The film composition depends on the operating temperature, and it seems insensitive on the used potential range. The operating temperature has a strong impact on the dissolution/deposition of alkaline fluorides on electrode surface upon cell cycling, as a result of which LiF is deposited instead of NaF at high temperatures, and the decomposed products become denser.

The best electrochemical performance (in terms of cycling stability and rate capability) is observed when *P*3-Na_2/3_Ni_1/3_Mg_1/6_Mn_1/2_O_2_ operates in hybrid Li/Na ion cell between 2.0 and 4.5 V using LiTFSI-Pyr_14_FSI electrolyte at 40°C. This performance of the oxide in hybrid Li/Na ion cell is better that that in single sodium ion cell due to dual intercalation of Li^+^ and Na^+^ ions. At 60°C, *P*3-Na_2/3_Ni_1/3_Mg_1/6_Mn_1/2_O_2_ delivers a higher capacity, but with a lower Coulombic efficiency owing to the massive cationic exchange reaction and electrode–electrolyte interaction. All the correlations between surface and bulk changes of *P*3-Na_2/3_Ni_1/3_Mg_1/6_Mn_1/2_O_2_ could be directed toward other materials with an aim to design such electrodes that exhibit electrochemical properties that are less sensitive toward the ambient temperature.

## Data Availability Statement

The raw data supporting the conclusions of this article will be made available by the authors, without undue reservation.

## Author Contributions

MK carried out the main experiment and performed material synthesis. KK and MK were involved in the discussion of the experimental results and revision of the manuscript. RS and EZ wrote the manuscript. MK and RS made the research plan. RS provided the financial support. All authors contributed to the article and approved the submitted version.

## Conflict of Interest

The authors declare that the research was conducted in the absence of any commercial or financial relationships that could be construed as a potential conflict of interest.
